# Acacetin reduces endoplasmic reticulum stress through the P‐eNOS/PERK signaling pathway to attenuate MGO‐induced vascular endothelial cell dysfunction

**DOI:** 10.1002/2211-5463.70004

**Published:** 2025-02-10

**Authors:** Zhen Zhang, Kaien Hu, Zhaohui Fang, Sihai Wang, Jie Chen, Dengke Yin, Caiyun Zhang, Gefei Ma

**Affiliations:** ^1^ School of Pharmacy Anhui University of Chinese Medicine Hefei Anhui China; ^2^ Department of Endocrine The First Hospital Affiliated to Anhui University of Chinese Medicine Hefei Anhui China; ^3^ Anhui Qimen Institute of Snakebite Huangshan China

**Keywords:** acacetin, calcium homeostasis, diabetic vascular diseases, endoplasmic reticulum stress, endothelial dysfunction, HUVECs apoptosis

## Abstract

Diabetic macrovascular disease is one of the most morbid and deadly complications of diabetes. Endothelial dysfunction plays a key role in diabetic macrovascular complications and endothelial cell apoptosis is one of the key indicators of endothelial dysfunction. Methylglyoxal (MGO), a highly reactive dicarbonyl compound generated during glycolysis, is related to the pathogenesis of cardiovascular diseases and may also promote endothelial dysfunction. Acacetin (ACA) is a naturally occurring flavonoid that can inhibit apoptosis, oxidative stress and inflammation to slow the progression of coronary heart disease; however, its effects on endothelial dysfunction are unknown. The present study investigated whether ACA may ameliorate MGO‐induced endothelial dysfunction in human umbilical vein endothelial cells. The results revealed that the viability and apoptosis of human umbilical vein endothelial cells induced by MGO decreased after ACA treatment, which was reflected in the expression levels of the apoptosis‐related proteins b‐cell lymphoma 2 (Bcl‐2)‐associated death, Bcl‐2‐associated x protein and Bcl‐2. Additionally, ACA downregulated the expression of key protein markers of MGO‐induced endoplasmic reticulum stress, physical evidence recovery kit, eukaryotic initiation factor 2 alpha, activating transcription factor 4 and C/EBP homologous protein, with which calcium inward currents may be closely related. ACA significantly downregulated the MGO‐induced expression of the cytosolic calcium channel proteins stromal interaction molecule 1, transient receptor potential canonical 1, ORAI calcium release‐activated calcium modulator 1, transient receptor potential vanilloid 1 and 4, and the trans‐endoplasmic reticulum membrane protein, transmembrane and coiled‐coil domains 1. Finally, ACA increased the expression of phosphorylated endothelial nitric oxide synthase (Ser1177), thus increasing the expression of nitric oxide in endothelial cells. Overall, acacetin could reduce endoplasmic reticulum stress through the phosphorylated‐endothelial nitric oxide/physical evidence recovery kit signaling pathway to attenuate MGO‐induced vascular endothelial cell dysfunction. These findings may hold potential for the use of acacetin in diabetic macrovascular complications.

## Graphical abstract

We investigated a potential protective effect of acacetin, a naturally occurring flavonoid, against methylglyoxal (MGO)‐induced endothelial dysfunction in human umbilical vein endothelial cells and its mechanism of action. Acacetin can could inhibit MGO‐induced intracellular calcium ion influx, enhance the expression levels of phosphorylated endothelial nitric oxide synthase (eNOS) and nitric oxide (NO). Simultaneously, it suppressed endoplasmic reticulum (ER) stress, reducing endothelial cell apoptosis and achieving effective prevention of endothelial dysfunction.

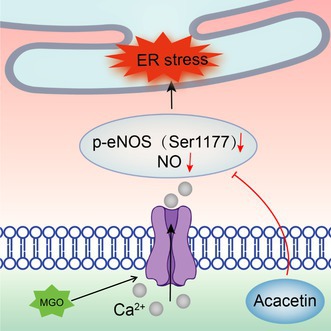

**Abbreviations**
4‐PBA: 4‐phenylbutiric acid
ACA: acacetin
AGE: advanced glycation end‐product
ANOVA: analysis of variance
ATF: activating transcription factor
BAD: Bcl‐2‐associated death
Bax: Bcl‐2‐associated x protein
Bcl‐2: b‐cell lymphoma 2
CHOP: C/EBP homologous protein
EC: endothelial cell
eIF2α: eukaryotic initiation factor 2 alpha
eNOS: endothelial nitric oxide synthase
ER: endoplasmic reticulum
ERS: endoplasmic reticulum stress
HUVECs: human umbilical vein endothelial cells
IRE1 alpha: inositol‐requiring protein 1 alpha
MGO: methylglyoxal
NAC: *N*‐acetylcysteine
NO: nitric oxide
ORAI1: ORAI calcium release‐activated calcium modulator 1
PERK: physical evidence recovery kit
qRT‐PCR: quantitative real‐time PCR
RAGE: receptor for advanced glycation end‐product
STIM1: stromal interaction molecule 1
TMCO1: transmembrane and coiled‐coil domains 1
TRPC1: transient receptor potential canonical 1
TRPV: transient receptor potential vanilloid

Diabetes is a heterogeneous disease related to glucose metabolism. The prevalence of diabetes around the world is ever‐increasing, with the number of people with this disease expected to grow from 537 million in 2021 to approximately 783 million by 2045 [1]. Diabetic macrovascular disease is a common complication of diabetes mellitus, which is caused by atherosclerosis and promotes events such as myocardial infarction, cerebrovascular disease and peripheral vascular disease. It is a major factor in the death and disability of diabetic patients [2, 3]. Dysfunction of endothelial cells (ECs), a major factor in the development of atherosclerosis, also influences the development of diabetic macrovascular lesions and is an independent risk factor for vascular disease, which is characterized by impaired vascular endothelial cell adhesion, vasodilatation and angiogenesis [4]. ECs may be affected by oxidative stress, which disrupts the cellular components integral to intracellular signal transduction pathways, leading to apoptosis [5, 6]. Methylglyoxal (MGO), a highly reactive dicarbonyl compound generated during glycolysis, forms an advanced glycation end‐product (AGE) adduct resulting from the fragmentation of propyl phosphate under hyperglycaemic conditions and subsequent activation of AGE receptors (RAGE) in endothelial cells [7, 8]. During glycolysis, MGO originates from two intermediates, phosphoglyceraldehyde and dihydroxyacetone phosphate, via the non‐enzymatic elimination of phosphate. MGO, as part of AGE, induces irreversible alterations in protein structure and function, potentially contributing to misfolding, which may result in cellular apoptosis [9]. MGO‐induced apoptosis, oxidative stress and AGE formation are particular episodes that induce vascular endothelial toxicity and lead to endothelial dysfunction [10]. MGO accumulates rapidly in a diversity of tissues and plays an important role in the etiology of many diabetic complications [11]. Consistently, previous studies have identified a novel proanthocyanidin from *Rhus tripartita* that mitigated MGO‐induced apoptosis in ECs under *in vitro* conditions [12].

Endoplasmic reticulum stress (ERS) is a key factor in triggering diabetes and diabetic complications. Disruption of vascular homeostasis as a result of endoplasmic reticulum damage, excessive activation and dysfunction all comprise a key pathogenic mechanism underlying vascular complications in diabetes mellitus [13]. MGO‐induced oxidative stress is linked to ERS through intracellular mediators, with MGO directly promoting the production of reactive oxygen species by interacting with its receptor and triggering ERS [10, 14]. Three main pathways are associated with ERS: protein kinase dsRNA‐activated protein kinase‐like endoplasmic reticulum kinase (PERK), inositol‐requiring protein 1 alpha (IRE1 alpha), and activation of transcription factors by transcription factor (ATF) 6 [15]. The activation of the PERK signaling pathway is considered the clearest marker of ERS and is involved in apoptosis [16]. Although the mechanism is not yet clear, studies have shown that ERS is strongly associated with intracellular calcium homeostasis. The endoplasmic reticulum (ER) serves as the primary location for intracellular calcium storage and is closely related to the homeostasis of intracellular Ca^2+^. Excessively high or low calcium levels in the ER can result in calcium signaling disorders, leading to abnormalities in cellular physiological functions and diseases [17]. Large quantities of Ca^2+^ flow into cells during cell injury and intracellular calcium overload increases inflammation and promotes apoptosis, necrosis and tissue damage [18]. MGO influences calcium channels in various cell types, with calcium overload and disrupted intracellular calcium homeostasis being significant contributors to cell apoptosis [19]. Research has shown that MGO decreases the viability of retinal pigment epithelial cells through ER stress‐dependent intracellular reactive oxygen species production and elevated intracellular calcium levels. In essence, MGO enhances intracellular calcium production [9]. Previous studies have indicated that acacetin (ACA) treatment alleviates FFA‐induced Ca^2+^ overload, suggesting that ACA protects cells from lipotoxicity by restoring Ca^2+^ homeostasis [20]. Endothelial nitric oxide synthase (eNOS) is a Ca^2+^/calmodulin‐dependent enzyme that is specifically expressed in endothelial cells and is responsible for nitric oxide (NO) production. The enzymatic activity of eNOS depends on the intracellular Ca^2+^ concentration and the interaction between eNOS and calmodulin [21]. Although the activation of eNOS is primarily regulated by various post‐translational modifications [22], which include protein phosphorylation and acetylation, phosphorylation of eNOS is paramount in facilitating its activation [23]. Ser1177 is a primary forward regulatory site for eNOS, for which phosphorylation enhances eNOS activity and NO production [24]. Studies have demonstrated that MGO inhibits insulin‐mediated activation of the eNOS/Akt pathway and NO release in endothelial cells [25]. Similarly, the protective effects of ACA on the vascular endothelium have been linked to the activation of the Akt/eNOS pathway [26]. ER stress significantly affects vascular lesions in diabetes mellitus and related cardiovascular diseases, and the inhibition of ERS in atherosclerotic lesions was found to slow the progression of atherosclerosis, thus protecting against diabetic vascular lesions [27].

Research has indicated that MGO elevates intracellular calcium levels in endothelial cells [28], with a rapid increase in intracellular calcium triggering eNOS activation at the same time as reducing NO production [29]. This process exacerbates ER stress and contributes to endothelial dysfunction [30].

Flavonoids have received widespread attention for their multiple biological activities, such as antimicrobial, antioxidant, antidepressant and anti‐diabetic properties [31]. The natural flavonoid luteolin has been shown to prevent MGO‐induced apoptosis through the activation of the mechanistic target of rapamycin/4E‐BP1 signaling pathway, a mechanism that may play a role in mitigating cognitive deficits associated with diabetes [32]. ACA is a natural flavonoid containing 5,7‐dihydroxy‐4′‐methoxyflavone [33], which is mainly found in some plants, such as *Robinia pseudoacacia* and *Pseudostellariae radix*. It is often used to prepare Chinese medicinal preparations, as well as folk herbal medicines. ACA has pharmacological effects, such as hypoglycemic effects, lipid regulation, cardioprotection, immunomodulation and antioxidant effects [34]. ACA can reduce the apoptosis of human umbilical vein endothelial cells (HUVECs) in an endothelial cell injury model, improve vasodilation function and alleviate endothelial dysfunction in insulin‐resistant rats [35]. ACA was found to decrease high glucose‐induced vascular endothelial damage through Sirt1‐mediated stimulation of Sirt3/AMPK activation and attenuate diabetes‐accelerated atherosclerosis [36]. The present study elucidated the protective effect of ACA against MGO‐induced endothelial dysfunction in HUVECs and its mechanism of action.

## Materials and methods

### Cell cultures and materials

HUVECs (M‐C1081; Shanghai Mcellbank Biotechnology Co., Ltd, Shanghai, China) were cultured in ECM (ScienCell, Carlsbad, CA, USA) under conditions of 37 °C, 5% CO_2_ and 95% humidity in an incubator (Thermo Fisher Scientific, Waltham, MA, USA). The ECM was supplemented with 5% fetal bovine serum, endothelial cell growth supplement and penicillin/streptomycin. The MGO was purchased from Macklin (Shanghai, China). Acacetin (DJ0037‐0020) (fineness ≥ 98%) was acquired from Dexter Biotech Ltd (Chengdu, China). The chemical composition for ACA is shown in Fig. S1A, as verified using NMR spectroscopy (Fig. S1B). The fineness in ACA determined using HPLC (Fig. S1C,D). To evaluate the cytoprotective role of ACA against MGO (1.8 mm)‐induced HUVECs, cells were prepared with ACA (3 μm) or *N*‐acetylcysteine (NAC) (5 mm) for 2 h, then incubated with MGO for 24 h, at least three replicate wells per group. The cells were harvested and extracted for further analysis.

### Cell viability assay

A CCK‐8 assay (CA1210; Solarbio, Beijing, China) was performed to determine cell viability. Cells (2 × 10^5^ cells·mL^−1^) were inoculated in 96‐well plates with 0.3, 1 or 3 μm ACA or 5 mm NAC in fresh medium for 2 h each, after which the cells were stimulated for 24 h with MGO (1.8 mm). Using an enzyme marker (Bio‐Rad, Hercules, CA, USA), cell viability was determined at 570 nm.

### Annexin‐V/propidium iodide staining and flow cytometry

HUVECs (5 × 10^5^ cells·mL^−1^) were inoculated in six‐well plates. Next, the cells were harvested and subsequently rinsed with chilled phosphate‐buffered saline before being analyzed with the aid of the Annexin V‐FITC Apoptosis Detection Kit (BB‐4101; Bestbio Co., Ltd, Shanghai, China). The proportion of apoptotic cells was assessed using flow cytometry (FACSCelesta; BD Biosciences, Franklin Lakes, NJ, USA).

### Fluorescence analysis of the cellular ER and calcium ion concentration

Endoplasmic reticulum localization was performed using ER‐Tracker Red (Beyotime, Shanghai, China) and the intracellular calcium concentration was detected using calcium fluorescent dye Fluo‐4AM (Beyotime). The cells were cultured in 24‐well plates (5 × 10^5^ cells·mL^−1^). The cells were incubated in phosphate‐buffered saline containing ER‐Tracker Red (1 μm) and Fluo‐4 AM (2 μm), rinsed with phosphate‐buffered saline to eliminate extracellular fluorescent dyes, and then observed under a confocal microscope (FV3000; Olympus, Tokyo, Japan).

### Chemiluminescence in measuring NO production

The concentration of NO released by HUVECs was quantified using an NO assay kit (Beyotime) strictly in accordance with the manufacturer's instructions. The total nitric oxide metabolites, including nitrate and nitrite, were quantified using chemiluminescence. The absorbance of the sample was measured at a wavelength of 540 nm and subsequently compared against a standard curve. The levels of nitrate and nitrite were normalized based on the protein content.

### Real‐time quantitative PCR

Total RNA was extracted using Trizol reagent (CWBIO, Jiangsu, China). The primer sequences were designed according to the GenBank cDNA sequences. A cDNA synthesis kit (TransGen, Shanghai, China) was employed to convert total RNA into cDNA through reverse transcription. A SYBR RT‐PCR Kit (TransGen) was used to analyze the mRNA levels of stromal interaction molecule 1 (STIM1), ORAI calcium release‐activated calcium modulator 1 (ORAI1), transient receptor potential vanilloid 1 and 4 (TRPV1 and TRPV4), transient receptor potential canonical 1 (TRPC1), transmembrane and coiled‐coil domains 1 (TMCO1) and glyceraldehyde‐3 phosphate dehydrogenase (internal control). An Opticon qPCR detection system (LightCycler96; Roche, Basel, Switzerland) was used for quantitative PCR analysis. The results were quantified via the 2−^ΔΔCT^ method. The sequences of the primers utilized were: glyceraldehyde‐3 phosphate dehydrogenase, 5′‐GAAGGTGAAGGTCGGAGTCAA‐3′ (forward) and 5′‐CTGGAAGATGGTGATGGGATTT‐3′ (reverse); b‐cell lymphoma 2 (Bcl‐2) cell death agonist (BAD), 5′‐AGCTCCGGAGGATGAGTGAC‐3′ (forward) and 5′‐ACCAGGACTGGAAGACTCGC‐3′ (reverse); STIM1, 5′‐AACAACCCTGGCATCCACTC‐3′ (forward) and 5′‐TCCATGTCATCCACGTCGTC‐3′ (reverse); ORAI1, 5′‐ACCTGTTTGCGCTCATGATC‐3′ (forward) and 5’‐GGACTCCTTGACCGAGTTGA‐3′ (reverse); TRPV1, 5′‐TCGGGGTCTTGGCCTATATT‐3′ (forward) and 5′‐ACCTCCAGCACCGAGTTCTT‐3′ (reverse); TRPV4, 5′‐ACCAGCCCCACATTGTCAAC‐3′ (forward) and 5′‐AGCGCATGCAGCACTGTGTT‐3′ (reverse); TRPC1, 5′‐CCTCTTGACAAACGGGGATT‐3′ (forward) and 5′‐ACCCGACATCTGTCCAAACC‐3′ (reverse); TMCO1, 5′‐AATCTGCTGGGAGATGACACC‐3′ (forward) and 5′‐GCAAGGCCGAGAATCTTCTGA‐3′ (reverse).

### Western blotting

Cells (1 × 10^5^ cells·well^−1^) were inoculated into six‐well plates. Cell lysates were collected using RIPA buffer enriched with protease and phosphatase inhibitors (Thermo Fisher Scientific). The lysates were subsequently subjected to centrifugation at 25 000 ***g*** for 15 min at 4 °C, following which the supernatants were harvested for subsequent analysis. The membranes underwent immunoblotting using primary antibodies at a dilution of 1:1000, followed by incubation with the appropriate secondary antibodies at a dilution of 1:5000. The blot was developed using the ECL substrate used for the assay (SparkJade, Shandong, China). imagej (NIH, Bethesda, MD, USA) was used for band size and density analysis of the protein blots. The antibodies used were: anti‐Bcl‐2‐associated x protein (Bax) (rabbit monoclonal antibody; CY5059; Abways Technology, Shanghai, China), anti‐Bcl2 (rabbit monoclonal antibody; CY6717; Abways Technology), anti‐PERK (rabbit monoclonal antibody; R25331; ZENBIO, SiChuan, China), anti‐phospho‐PERK (rabbit monoclonal antibody; 340 846; ZENBIO), anti‐eukaryotic initiation factor 2 alpha (eIF2α) (rabbit monoclonal antibody; R24185; ZENBIO), anti‐phospho‐eIF2α (rabbit monoclonal antibody; R22946; ZENBIO), anti‐ATF4 (rabbit monoclonal antibody; R381426; ZENBIO), anti‐C/EBP homologous protein (CHOP) (rabbit monoclonal antibody; CY6694; Abways Technology, Shanghai, China), anti‐IRE1α (rabbit polyclonal antibody; BD‐PN5428; Biodragon, JiangSu, China), anti‐phospho‐IRE1α (rabbit monoclonal antibody; CY5605; Abways Technology, Shanghai, China); anti‐eNOS (rabbit polyclonal antibody; CY3412; Abways Technology) and anti‐phospho‐eNOS (Ser1177) (rabbit monoclonal antibody; 310 209; ZENBIO).

### Statistical analysis

Multiple groups were compared for statistical differences by one‐way analysis of variance (ANOVA). An unpaired Student's *t*‐test was used for comparisons between two groups. Data are presented as the mean ± SE of the three experiments. *P* < 0.05 was considered statistically significant.

## Results

### ACA improved the viability of MGO‐treated HUEVCs

**Fig. 1:**
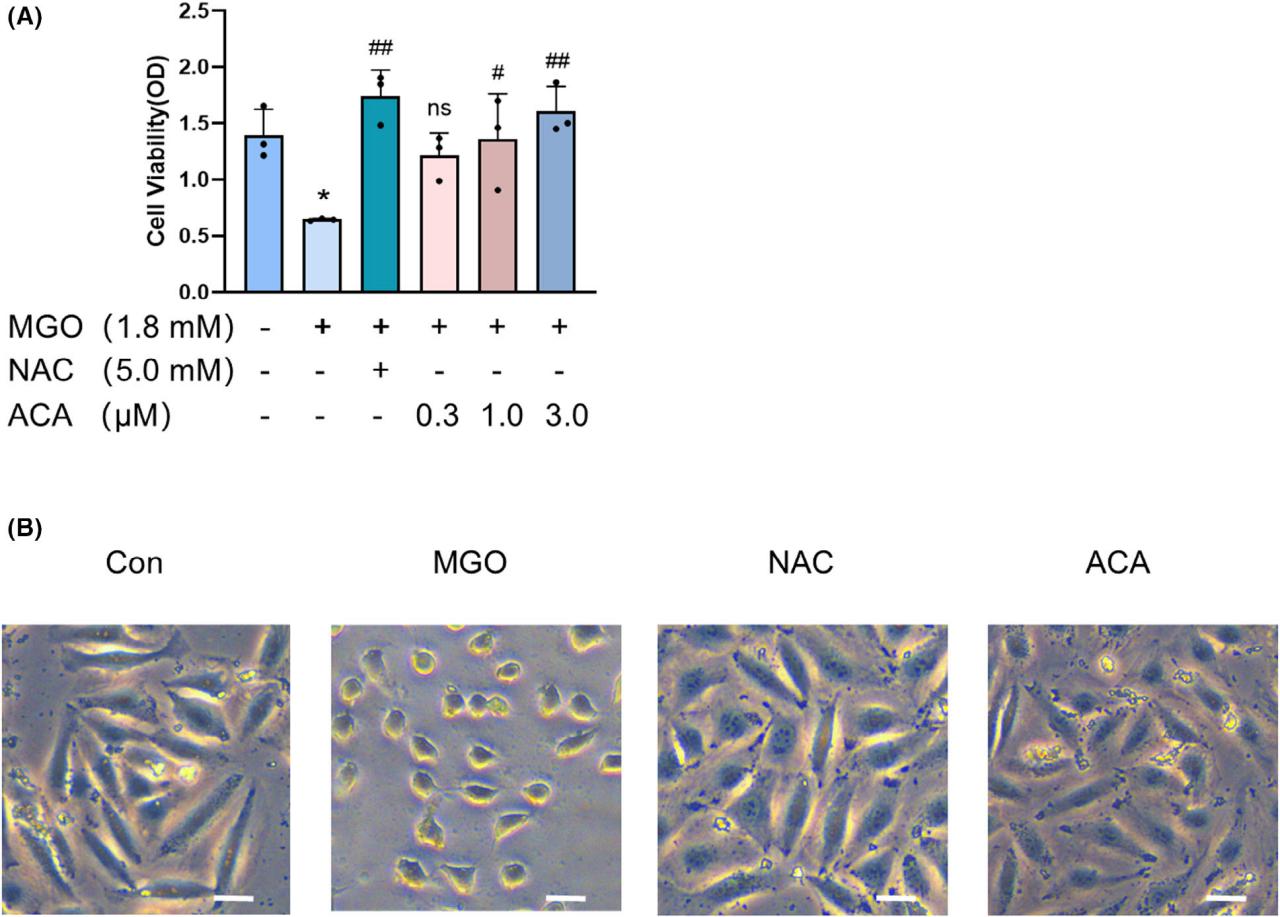
The protective effect of acacetin on MGO‐induced HUVECs viability. (A) Cell viability of HUVECs. Because exposure of HUVECs to MGO for 24 h significantly decreased cell viability, adding ACA (0.3–3 μm) increased the viability of MGO‐induced HUVECs in a dose‐dependent manner. Bars indicate the mean ± SE (*n* = 3). *P < 0.05 vs. normal control; ^#^ *P* < 0.05 and ^##^ *P* < 0.01 vs. MGO alone treatment group; n.s., not significant. Data were analyzed using one‐way ANOVA. (B) Representative phenotypes of MGO‐treated HUVECs. Scale bar = 10 μm. The data are expressed as the mean ± SD (*n* = 3).

To ascertain the protective effect of ACA in MGO‐induced damage to vascular endothelial cells, we performed a CCK‐8 assay to detect cellular activity. Exposure of HUVECs to MGO for 24 h significantly decreased cell viability and adding ACA (0.3–3 μm) increased the viability of MGO‐induced HUVECs in a dose‐dependent manner. When the concentration of ACA was increased to 3 μm, which significantly increased the viability of MGO‐induced HUVECs, 3 μm ACA was used as the experimental concentration to confirm its protective effect against damage to HUVECs. The results also indicated that 3 μm ACA had an effect comparable to that of 5 mm NAC on MGO‐induced cell damage (Fig. 1A). Microscopic observations revealed morphological changes in the cells as well. Compared to the control group, the MGO‐induced HUVECs were atrophied and degenerated. By contrast, the morphology of the ACA‐treated cells did not change significantly. The morphology of cells within the NAC group closely resembled that of the normal group (Fig. 1B). The findings suggested that ACA improved the viability and morphology of MGO‐induced HUVECs.

### ACA inhibited the apoptosis of MGO‐stimulated HUVECs

**Fig. 2:**
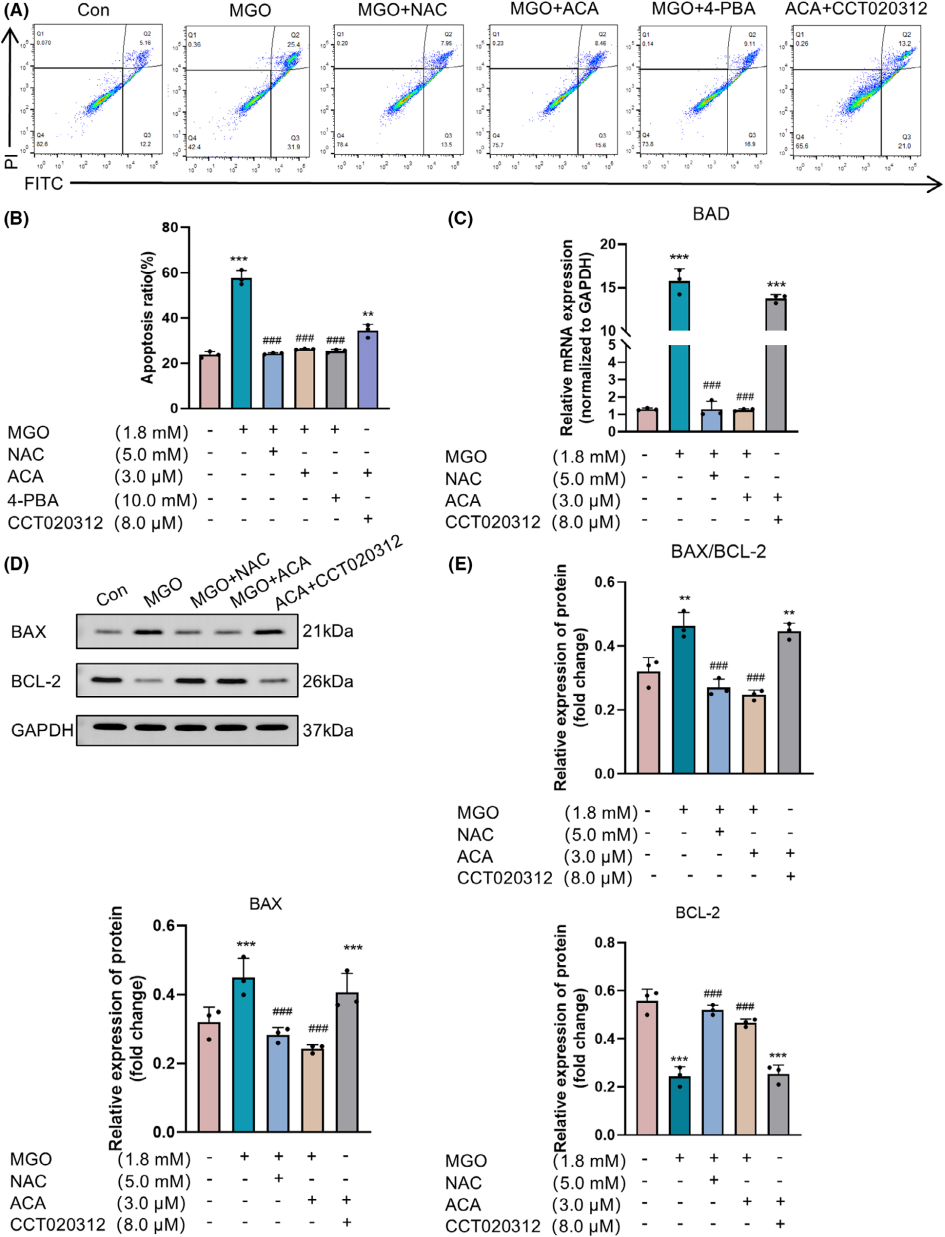
The protective effect of acacetin on MGO‐induced HUVECs apoptosis. (A) Annexin V‐FITC/propidium iodide assay showing the protective effect of acacetin against MGO‐induced apoptosis in HUVECs (*n* = 3). (B) Apoptosis rate. The bars indicate the mean ± SE (*n* = 3). **P < 0.01 and ***P < 0.001 vs. the normal control group; ^###^ *P* < 0.001 vs. the MGO alone treatment group. (C) mRNA expression of the apoptotic protein BAD measured by qRT‐PCR. Bars indicate the mean ± SE (*n* = 3). ***P < 0.001 vs. the normal control group; ^###^ *P* < 0.001 vs. the MGO alone treatment group. Data were analyzed using one‐way ANOVA. (D) Immunoblot analysis of proteins extracted from cells to confirm Bax and Bcl‐2 protein levels. (E) Protein levels of Bax and Bcl‐2 in HUVECs. Scale bars indicate the mean ± SE (*n* = 3), **P < 0.01 and ***P < 0.001 vs. the normal control group; ^###^ *P* < 0.001 vs. the MGO alone treatment group. Data were analyzed using one‐way ANOVA.

The investigation examined the protective effect of ACA against apoptosis induced by MGO. We evaluated the degree of apoptosis induced by MGO in HUVECs using flow cytometry. Compared with normal conditions, MGO stimulation increased apoptosis, which was significantly inhibited by ACA treatment. Treatment with the ER stress inhibitor 4‐phenylbutiric acid (4‐PBA) and the antioxidant NAC similarly exhibits inhibitory effects on the cells (Fig. 2A,B). BAD, a BH3‐only protein within the Bcl‐2 family, plays a pivotal role in the regulation of apoptosis [37]. The quantitative real‐time PCR (qRT‐PCR) results indicated that the expression of BAD increased after MGO stimulation compared to that recorded in the normal group and that this expression of BAD was inhibited by ACA treatment (Fig. 2C). Western blotting analysis was conducted to ascertain if apoptosis induced by MGO in HUVECs was linked to changes in the expression of antiapoptotic Bcl‐2 and proapoptotic Bax proteins. Compared with normal control cells, MGO‐stimulated cells exhibited a marked reduction in the expression of the antiapoptotic protein Bcl‐2 and a substantial elevation in the expression of the proapoptotic protein Bax. ACA inhibited the increase in Bax, enhanced the reduction in Bcl‐2 and elevated the reduction in the Bax/Bcl‐2 ratio. NAC treatment had the same effect on the cells (Fig. 2D,E). These results indicate that ACA protects HUVECs from MGO injury to vascular endothelial cells by inhibiting apoptosis.

### ACA reduced ER stress in MGO‐treated HUEVCs

**Fig. 3:**
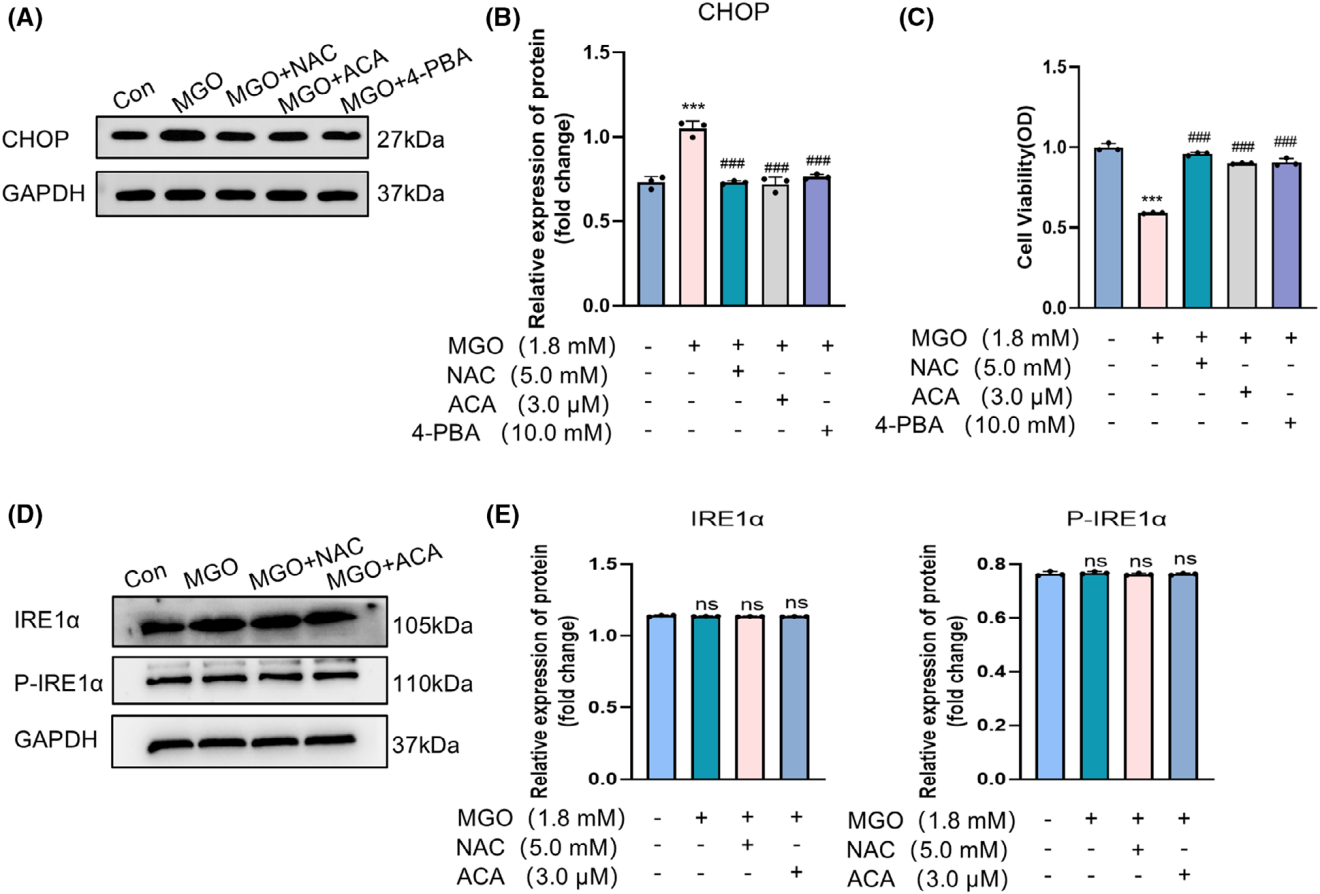
Acacetin mitigates the ER stress response induced in MGO‐treated HUVECs. (A) Proteins extracted from HUVECs were analyzed by immunoblotting to confirm CHOP protein levels (*n* = 3). (B) Protein levels of CHOP in HUVECs were analyzed. Quantitative data are presented as the mean ± SE (*n* = 3). ***P < 0.001 vs. the normal control group; ^###^ *P* < 0.001 vs. the MGO‐alone treatment group. Data were analyzed using one‐way ANOVA. (C) The effect of ER stress inhibitor 4‐PBA on HUVECs viability was assessed. Bars indicate the mean ± SE (*n* = 3). ***P < 0.001 vs. the normal control group; ^###^ *P* < 0.001 vs. the MGO alone treatment group. Data were analyzed using one‐way ANOVA. (D) Immunoblot analysis of proteins extracted from HUVECs was performed to confirm IRE1α and P‐IRE1α protein levels (*n* = 3). (E) Protein levels of IRE1α and P‐IRE1α in HUVECs. Bars indicate the mean ± SE (*n* = 3), n.s., not significant. Data were analyzed using one‐way ANOVA.

To evaluate the protective efficacy of ACA against ERS in MGO‐induced HUVECs, CHOP, a key biomarker of ERS, was quantified through western blot analysis. The findings revealed that MGO‐induced cells exhibited elevated CHOP protein expression compared to the normal group. However, ACA treatment significantly inhibited this upregulation, an effect similarly observed with NAC treatment. To further investigate the relationship between ACA and ER stress in MGO‐induced cells, HUVECs were treated with the ER stress inhibitor 4‐PBA, which also effectively suppressed MGO‐induced CHOP upregulation (Fig. 3A,B). Additionally, MGO exposure markedly reduced cell viability, whereas ACA treatment significantly restored it. A comparable protective effect was noted with 4‐PBA and NAC treatments (Fig. 3C). These findings suggest that MGO induces ER stress in HUVECs and ACA exerts a protective effect by mitigating this stress.

**Fig. 4:**
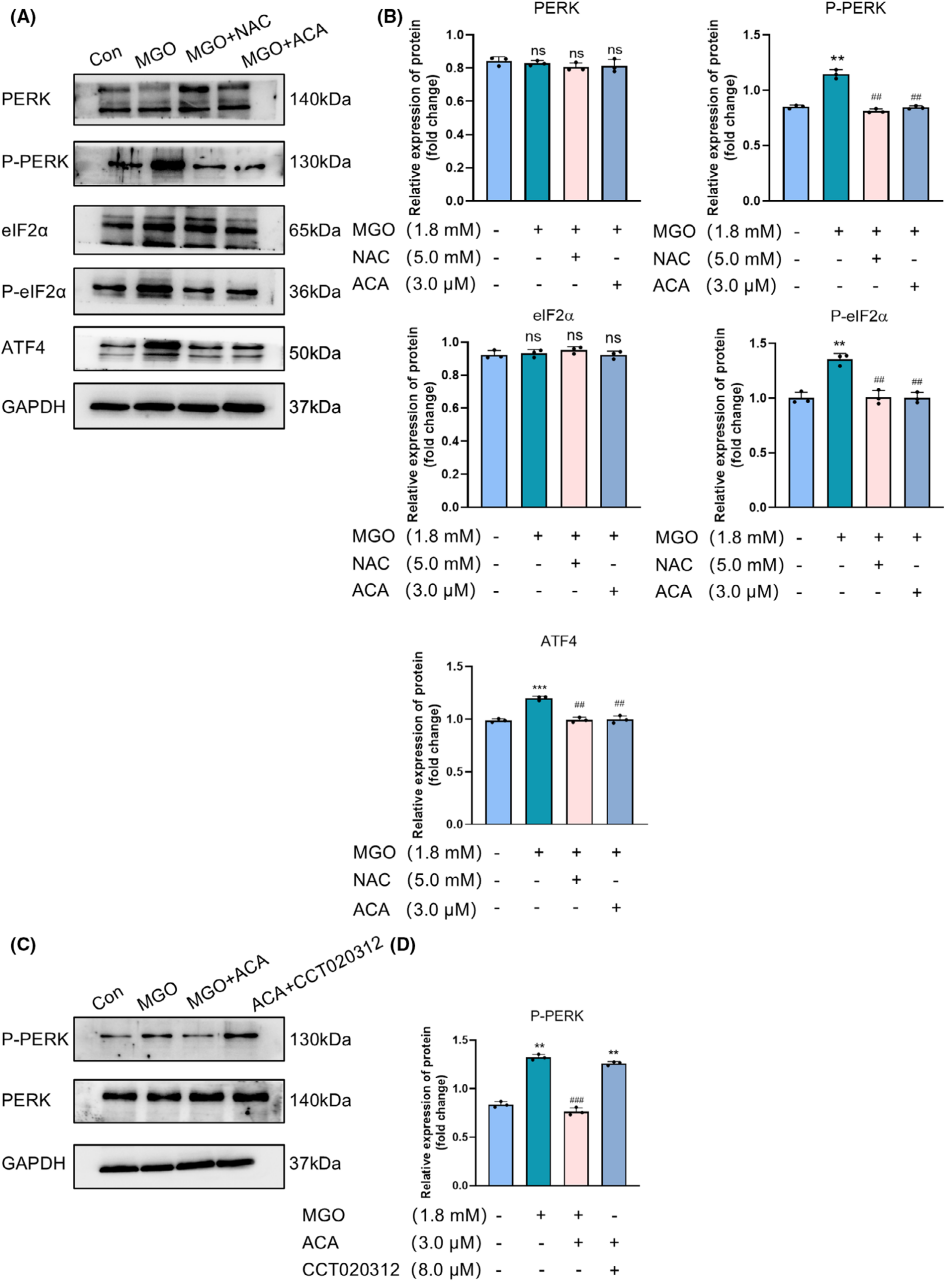
Acacetin protects the MGO‐induced PERK signaling pathway in HUVECs. (A) Immunoblot analysis of PERK, P‐PERK, eIF2α, P‐eIF2α and ATF4 protein expression (*n* = 3). (B) Bars indicate the mean ± SE (*n* = 3). **P < 0.01 and ***P < 0.001 vs. the normal control group; ^##^ *P* < 0.01 and ^###^ *P* < 0.001 vs. the MGO alone treatment group; n.s., not significant. Data were analyzed using one‐way ANOVA. (C) Immunoblot analysis of PERK protein levels after treatment with the agonist CCT020312 (*n* = 3). (D) Bars indicate the mean ± SE (*n* = 3), **P < 0.01 vs. the normal control group; ^###^ *P* < 0.001 vs. the MGO alone treatment group. Data were analyzed using one‐way ANOVA.

To investigate the effect of ACA on ER stress mediated by the PERK signaling pathway, key proteins PERK, eIF2α and ATF4 were analyzed as markers of ER stress using western blotting. MGO‐induced cells exhibited increased expression of P‐PERK, P‐eIF2α and ATF4 proteins compared to normal controls, whereas ACA treatment significantly inhibited this upregulation. A similar inhibitory effect was observed with NAC treatment (Fig. 4A,B). Further analysis showed that CCT020312, a selective activator of eIF2AK3/PERK, enhanced PERK phosphorylation and counteracted the protective effect of ACA on MGO‐induced cells (Fig. 4C,D). Additionally, western blot analysis of the anti‐apoptotic Bcl‐2 and pro‐apoptotic Bax proteins revealed that, in the ACA + CCT020312 group, Bax expression and the Bax/Bcl‐2 ratio were significantly elevated, whereas Bcl‐2 expression was reduced compared to the normal group (Fig. 2D,E). qRT‐PCR results demonstrated increased BAD expression in the ACA + CCT020312 group relative to normal controls (Fig. 2C). Flow cytometry further confirmed enhanced apoptosis in ACA + CCT020312‐treated cells (Fig. 2A,B). Examination of the role of IRE1α in the ER stress pathway revealed no significant change in IRE1α expression after 24 h of treatment with MGO and ACA (Fig. 3D,E). These findings suggest that ACA alleviates ER stress through the PERK pathway, mitigating MGO‐induced dysfunction in HUVECs.

### ACA reduced ERS and calcium ion accumulation in MGO‐treated HUEVCs

**Fig. 5:**
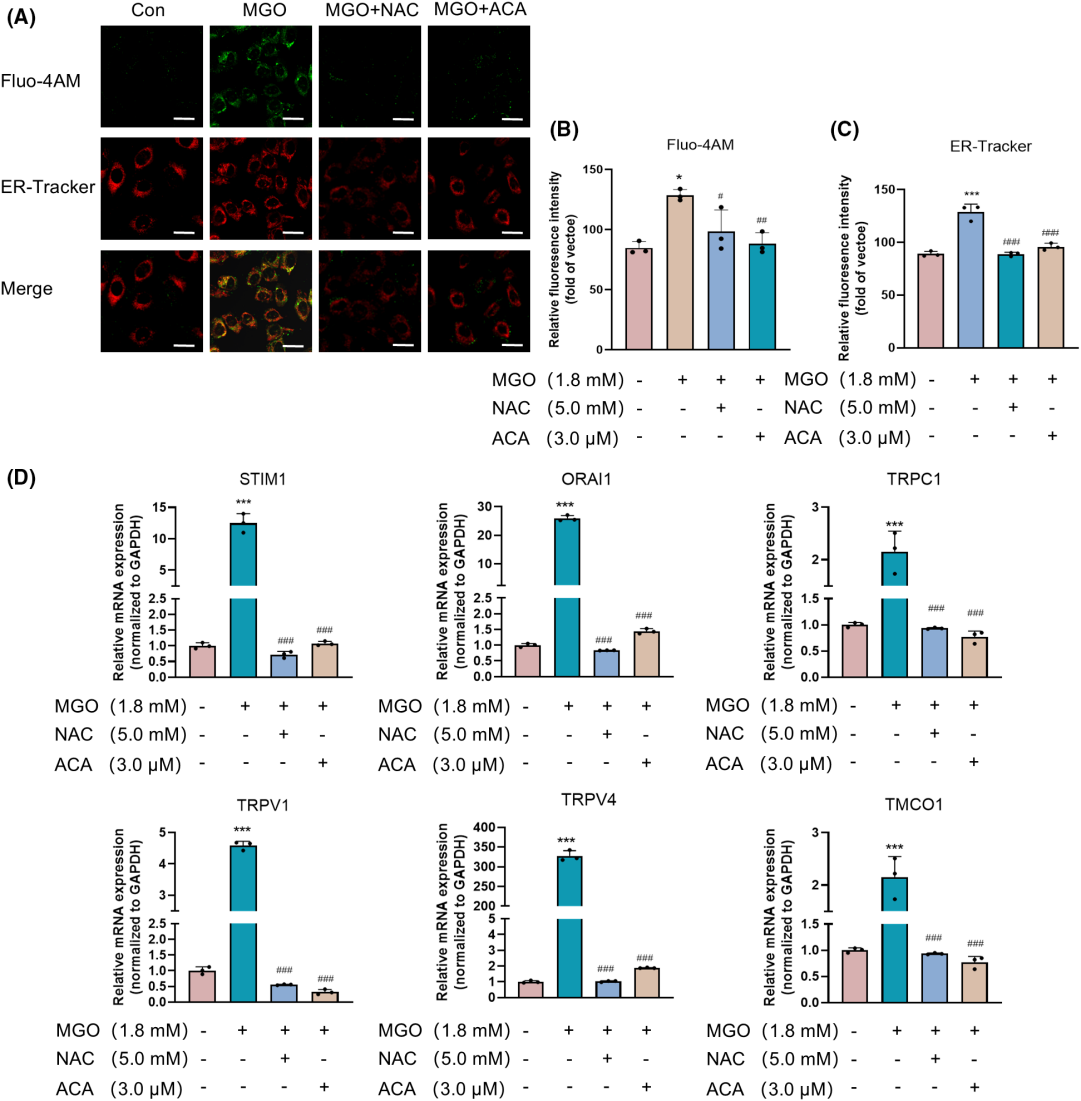
Acacetin relieves MGO‐induced endoplasmic reticulum stress and calcium overload in HUEVCs. (A) Detection of ER stress in HUEVCs via Fluo‐4 AM and ER‐Tracker. Scale bar = 10 μm (*n* = 3). (B) Because ERS alters intracellular Ca^2+^ homeostasis, changes in the intracellular Ca^2+^ concentration were also monitored via the fluorescent calcium indicator Fluo‐4AM. The bars indicate the mean ± SE (*n* = 3). *P < 0.05 vs. the normal control group; ^#^ *P* < 0.05 and ^##^ *P* < 0.01 vs. the MGO alone treatment group. Data were analyzed using one‐way ANOVA. (C) ER‐Tracker Red was used to assess whether the addition of ACA to MGO‐induced HUVECs reduces ERS. Bars indicate the mean ± SE (*n* = 3). ***P < 0.001 vs. the normal control group; ^###^ *P* < 0.001 vs. the MGO alone treatment group. (D) qRT‐PCR was performed to detect mRNA expression of the calcium overload proteins STIM1, TRPC1, ORAI1, TMCO1, TRPV1 and TRPV4. Bars indicate the mean ± SE (*n* = 3). ***P < 0.001 vs. the normal control group; ^###^ *P* < 0.001 vs. the MGO alone treatment group. Data were analyzed using one‐way ANOVA.

We used ER‐Tracker Red to assess whether the addition of ACA reduces ERS in HUVECs. These results indicate that ERS can also change the state of the ER. Compared to the control group, red fluorescence in the MGO‐treated group was greater, reflecting a significant improvement in ER permeability and the aggravation of ER stress. Adding ACA effectively decreased ER permeability, indicating that ACA may inhibit the development of ER stress. Similar effects were also observed in response to NAC treatment (Fig. 5A,B). These results indicated a protective effect of ACA against ERS in MGO‐induced HUVECs.

Because ERS alters intracellular Ca^2+^ homeostasis, changes in the intracellular Ca^2+^ concentration were also monitored via the fluorescent calcium indicator Fluo‐4 AM. In comparison with the control group, the fluorescence of Fluo‐4 AM‐treated cells treated with MGO was significantly greater, whereas ACA treatment alleviated Ca^2+^ overload in these cells. A similar effect was also observed after NAC treatment (Fig. 5A,C). The qRT‐PCR analysis results indicated that the expression levels of the calcium channel proteins STIM1, TRPC1, ORAI1, TRPV1 and TRPV4 and the transmembrane protein TMCO1 were significantly greater than that in the control group. Additionally, the upregulation of these proteins was inhibited by ACA and NAC (Fig. 5D). The findings suggest that ACA has the potential to diminish the buildup of Ca^2+^ in HUVECs triggered by MGO and to decrease the expression of calcium channel proteins.

### ACA promoted NO production and P‐eNOS expression in MGO‐induced HUEVCs

**Fig. 6:**
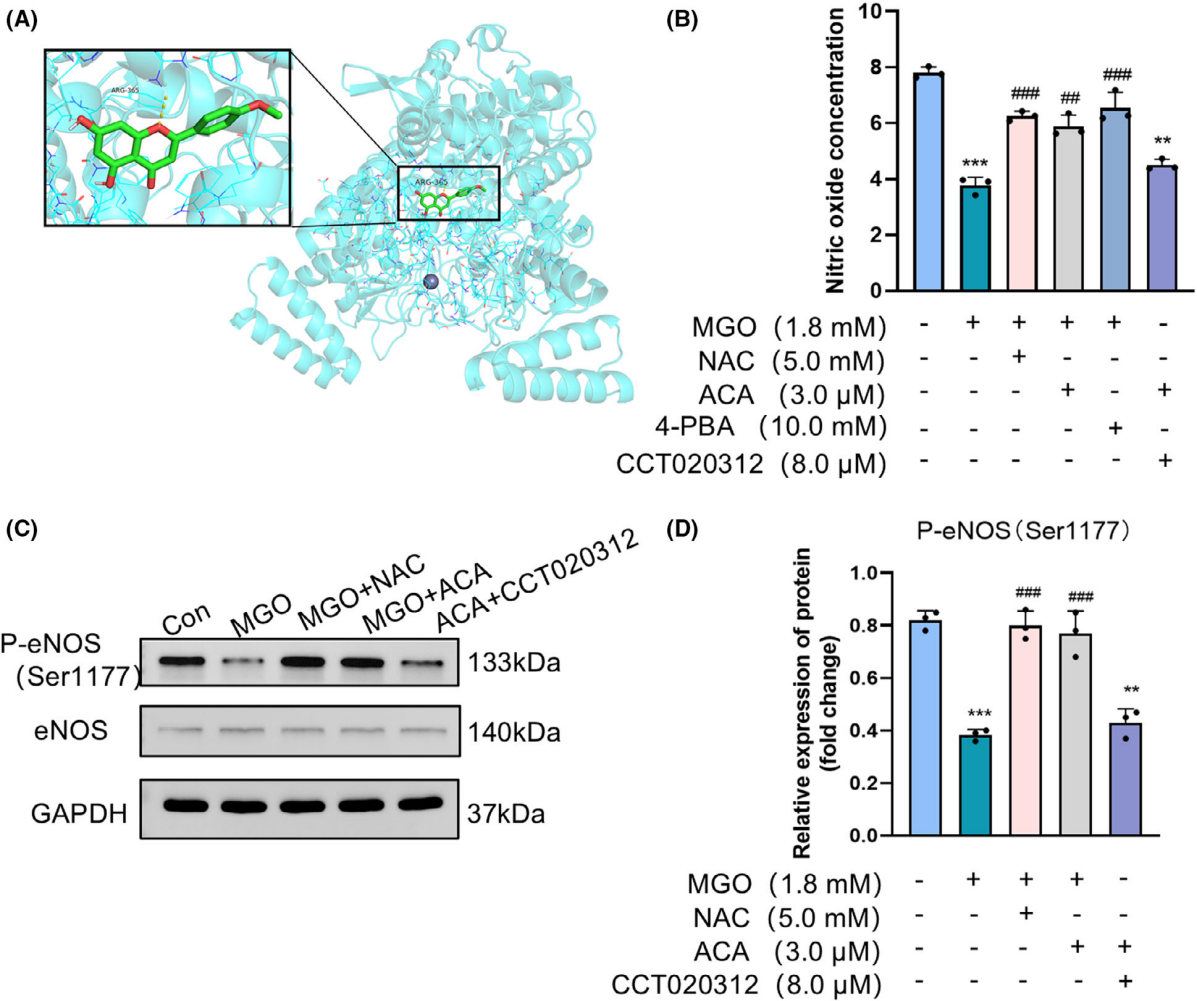
Acacetin decreases the MGO‐induced increase in NO concentration and protein expression of eNOS/P‐eNOS (Ser1177) in HUVECs. (A) Prediction of the interaction of eNOS with acacetin. The blue backbone represents chrysin, the red line represents the oxygen atom, the green helix represents eNOS and the yellow dashed line represents the hydrogen bond between the chrysin and eNOS amino acids. (B) The degree of phosphorylation of Ser1177 is positively correlated with the amount of NO released. Therefore, the concentration of NO in HUVECs was measured. Bars indicate the mean ± SE (*n* = 3). **P < 0.01 and ***P < 0.001 vs. the normal control group; ^##^ *P* < 0.01 and ^###^ *P* < 0.001 vs. the MGO alone treatment group. Data were analyzed using one‐way ANOVA. (C) Immunoblot analysis of eNOS and P‐eNOS (Ser1177) protein expression (*n* = 3). (D) Bars indicate the mean ± SE (*n* = 3). **P < 0.01 and ***P < 0.001 vs. the normal control group; ^###^ *P* < 0.001 vs. the MGO alone treatment group. Data were analyzed using one‐way ANOVA.

Although ACA has hypoglycemic, cardioprotective and antioxidative effects, research on its direct targets is limited. To identify potential direct targets of ACA, we used Swiss‐Target Prediction (http://www.swisstargetprediction.ch) to predict approximately 300 possible targets, among which eNOS scored the highest. Some studies have shown that eNOS can protect against atherosclerosis [38] and diabetes‐related complications [39]. Thus, we anticipated a direct interaction between eNOS and ACA, employing discovery studio (https://www.3ds.com/products/biovia/discovery‐studio) for our analysis (Fig. 6A). The affinity value of the interaction was −9.4 kcal·mol^−1^ (Fig. S2), a figure lower than −5 kcal·mol^−1^, indicating that eNOS binds strongly to ACA. Ser1177 serves as a key positive regulatory site for eNOS, and its phosphorylation increases eNOS activity [35]. The western blotting assays demonstrated that the expression of phosphorylated eNOS (Ser1177) in HUVECs, induced by MGO, was markedly reduced compared to the control group. By contrast, treatment with ACA and NAC resulted in an upregulation of phosphorylated eNOS expression in HUVECs. Furthermore, HUVECs treated in the ACA + CCT020312 group demonstrated inhibited phosphorylation of eNOS at Ser1177 (Fig. 6C,D). The findings suggest that ACA has the potential to enhance the expression of phosphorylated eNOS within MGO‐induced HUVECs.

The degree of phosphorylation of Ser1177 is positively correlated with the amount of NO released [40]. The findings revealed that NO activity was significantly reduced in MGO‐induced HUVECs compared to the control group, whereas ACA treatment effectively increased NO activity. A similar trend was observed with the use of the ER stress inhibitor 4‐PBA and the antioxidant NAC. However, NO production decreased in cells treated with ACA + CCT020312 (Fig. 6B). These results indicated that ACA could increase NO activity in MGO‐induced HUVECs.

## Discussion

Diabetes is a metabolic and inflammatory disease characterized by hyperglycemia. Its growth is an epidemic. The high prevalence of diabetes and its high disability and mortality rates have greatly increased the economic burden on families and society [41]. Hyperglycemia significantly increases the risk of cardiovascular disease. The progression of cardiovascular disease is driven by hyperglycemia, leading to the generation of dicarbonyl compounds, which are active glucose metabolites that interact with protein residues to form AGEs [42]. MGO is the main precursor for the formation of AGEs and is the most reactive dicarbonyl group. It is associated not only with hyperglycemia in patients with diabetes, but also with other risk factors for vascular complications of diabetes, such as hypertension, dyslipidemia and obesity. These findings indicate that MGO plays a key role in developing vascular complications in diabetes [20]. MGO promotes endothelial dysfunction, microvascular complications and macrovascular complications such as atherosclerosis and impaired hemodialysis [43]. Previous studies have shown that MGO induces apoptosis in various endothelial cells, including those in the brain and intestine, and disrupts endothelial barrier function [10, 44].

Additionally, MGO has been reported to elevate intracellular Ca^2+^ levels in endothelial cells such as HUVECs [28]. A rapid increase in intracellular calcium stimulates the calcium‐calmodulin complex to bind to the calmodulin‐binding domain of the enzyme, activating eNOS and reducing NO production. Furthermore, endothelial calcium signaling acts as an upstream regulator for eNOS phosphorylation at Ser1177 [29]. Activation of the AMPK/eNOS signaling pathway has been associated with increased ER stress and oxidative stress, ultimately contributing to endothelial dysfunction [30].

Flavonoids have various biological activities, including antioxidant, anti‐inflammatory and anticancer activities. They also inhibit oxidative stress and induce apoptosis [45]. Previous research has examined the MGO‐scavenging activities of various flavonoids and their subcomponents, identifying key structural requirements and additive effects that enable flavonoids to neutralize MGO [46]. The flavonoid apigenin has been reported to capture MGO and form an adduct, thus inhibiting the formation of AGEs and exerting protective effects on HUVECs through the induction of the ERK/Nrf2 pathway [47]. Additionally, other flavonoids and their derivatives, including luteolin [13], polydatin (a resveratrol precursor) [48] and the resveratrol derivative pterostilbene [49], have been shown to prevent MGO‐induced apoptosis in HUVECs. These findings suggest that identifying drugs derived from flavonoids or their derivatives represents a promising strategy for mitigating endothelial dysfunction by targeting MGO‐induced cellular apoptosis. ACA is a flavonoid found in many plants and in many dietary sources. It also has various biological activities, including anti‐inflammatory, antioxidant and antiobesity effects [50]. The anti‐inflammatory and antioxidant properties of ACA may decrease atherosclerosis induced by oxidative stress and reduce endothelial dysfunction in the human endothelial cell line EA.hy926. Similarly, ACA can inhibit the apoptosis of cardiomyocytes by reducing the release of inflammatory cytokines [51]. Furthermore, our result on the CCK‐8 results indicated that MGO significantly reduced the viability of HUVECs and increased their apoptosis, whereas ACA protected HUVECs from the MGO‐induced decrease in cell viability (Fig. 1A). To examine the protective impact of ACA on apoptosis induced by MGO, we conducted Annexin V‐FITC/propidium iodide assays via flow cytometry. We found that MGO decreased cell viability in association with increased apoptosis, which was significantly inhibited after ACA was added (Fig. 2A,B). Consistent with these results, exposure of HUVECs to MGO decreased Bcl‐2 expression and increased Bax expression. By contrast, ACA treatment decreased the increase in Bax protein expression, increased Bcl‐2 protein expression, and inhibited the increase in the Bax/Bcl‐2 ratio, confirming that ACA is resistant to MGO‐induced apoptosis (Fig. 2D,E). These results indicated that ACA effectively prevented the apoptosis of HUVECs.

ERS promotes the development of endothelial dysfunction by perturbing the vasoactive homeostasis of endothelial cells. ERS may initially cause endothelial dysfunction and induce endothelial cell apoptosis, thus promoting atherosclerosis [52]. Sustained activation of the intracellular PERK pathway ultimately results in the overexpression of CHOP, leading to apoptosis [53]. MGO has been identified as a factor that induces ERS and apoptosis in endothelial cells through the AMPK pathway, contributing to aortic endothelial dysfunction [15]. Additionally, MGO‐induced endothelial dysfunction in the brain has been linked to oxidative stress and ER stress mechanisms [54]. Research indicates that flavonoids have the potential to inhibit ER stress and oxidative stress, thus improving endothelial dysfunction associated with obesity and diabetes [30]. These observations align with the findings that ACA provides a protective effect against ERS in MGO‐induced HUVECs. The results indicated that the protein expression of the ER stress marker CHOP was elevated in MGO‐induced HUVECs, and ACA treatment effectively suppressed the upregulation of CHOP. Furthermore, treatment with the ERS inhibitor 4‐PBA also demonstrated effectiveness in reducing MGO‐induced CHOP upregulation (Fig. 3A,B).

We found that ACA inhibited MGO‐induced protein expression of P‐PERK, P‐eIF2α and ATF4 regulated MGO‐induced endothelial ERS in endothelial cells (Fig. 4A,B). We determined whether ACA can act through the PERK pathway via the PERK agonist CCT020312. The results indicated that CCT020312 significantly weakened the protective effect of ACA on MGO‐induced cells (Fig. 4C,D). Further research results demonstrate that ACA combined with CCT020312 increased Bax expression at the same time as decreasing Bcl‐2 expression (Fig. 2D,E), leading to enhanced apoptosis (Fig. 2A,B). Similarly, ACA + CCT020312‐treated HUVECs showed inhibition of eNOS (Ser1177) phosphorylation expression (Fig. 6C,D). The rate of cellular NO production was also evaluated in ACA + CCT020312‐treated HUVECs, revealing a reduction in cells exposed to ACA + CCT020312 (Fig. 6B). Tests conducted on the IRE1α pathway indicated that the changes in phosphorylated IRE1α expression in cells incubated with MGO and ACA were not significant (Fig. 3D,E). These findings suggest that the inhibition of MGO‐induced apoptosis in HUVECs by ACA is likely associated with the PERK pathway of endoplasmic reticulum stress.

Endothelial dysfunction in diabetes mellitus is closely associated with disruptions in intracellular calcium homeostasis. Ca^2+^ is a major trigger of endothelial cell apoptosis [55]. The increase in the intracellular Ca^2+^ concentration is derived mainly from the inward flow of extracellular Ca^2+^ and the release of intracellular calcium stores. The transmembrane protein TMCO1 in the ER senses the concentration of calcium in the ER and monitors it in real time to maintain homeostasis of the calcium pool [56]. The associated proteins TRPC1, Orai1, STIM1, TRPV4 and TRPV1 in the cytosolic calcium channels maintain intracellular Ca^2+^ homeostasis [57]. MGO can induce intracellular Ca^2+^ mobilization from ER stores, triggering ERS in lens epithelial cells [20]. MGO treatment led to increased intracellular calcium accumulation in HUVECs, subsequently inducing apoptosis [28]. ACA can regulate calcium homeostasis in pancreatic cells, thus reducing apoptosis as a result of ERS [58]. Another study reported that decreasing the influx of calcium ions can promote the recovery of endothelial cell function [59]. Therefore, we investigated the relationship between ACA and the inward flow of calcium; the results revealed that ACA inhibited the expression of STIM1, Orai1, TRPC1, TRPV1, TRPV4 and TMCO1 and reduced the intracellular Ca^2+^ concentration in MGO‐induced HUVECs (Fig. 5D). The mutual relationship between Ca^2+^ channel proteins and endoplasmic reticulum stress remains insufficiently understood, necessitating further exploration in subsequent research.

ERS in endothelial cells can directly reduce the expression and phosphorylation levels of eNOS and may further downregulate NO production. Vascular endothelial function is impaired when NO bioavailability decreases [60]. Studies have reported that treatment of rat thoracic aortic rings with MGO resulted in decreased levels of eNOS and its phosphorylated form, P‐eNOS (Ser1177), indicating that MGO may contribute to endothelial dysfunction by reducing P‐eNOS (Ser1177) levels [61]. Additionally, MGO has been shown to influence eNOS‐related functions in endothelial cells, causing uncoupling and hypophosphorylation of eNOS, which increases superoxide production and adversely affects endothelial cell function [26]. Moreover, ACA has been observed to prevent endothelial dysfunction in hypertension by regulating mitochondrial function via activation of the Akt/eNOS pathway [62]. The findings in the present study revealed that MGO‐induced HUVECs exhibited a reduction in eNOS (Ser1177) phosphorylation and NO expression. However, ACA treatment effectively increased the expression of phosphorylated eNOS (Ser1177), thereby enhancing NO production in endothelial cells (Fig. 6B–D).

**Fig. 7:**
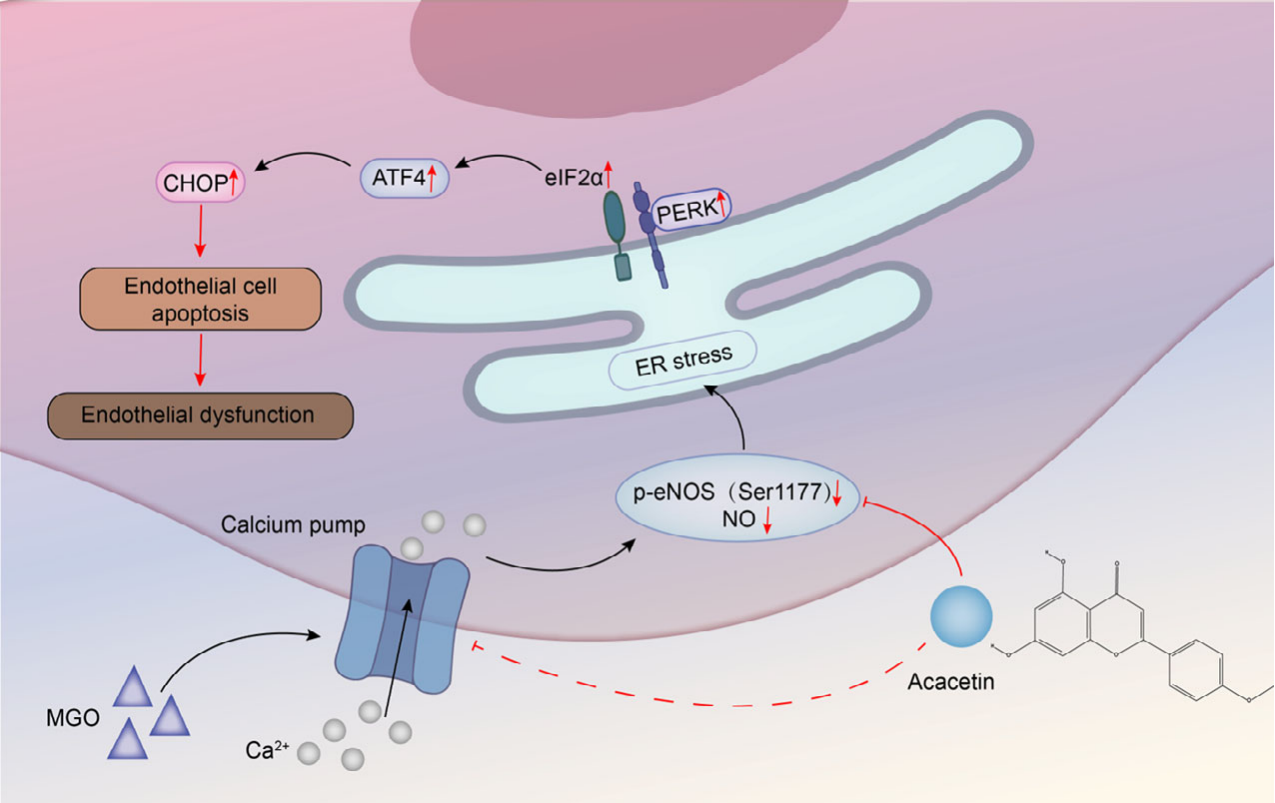
The present study demonstrated that acacetin attenuates the effects of MGO‐induced vascular endothelial cell dysfunction. The main mechanism is that acacetin regulates calcium overload in MGO‐induced cells; upregulates P‐eNOS (Ser1177) and NO; decreases the expression of PERK, eIF2α, ATF4 and CHOP, which are key protein markers of endoplasmic reticulum stress; and reduces endothelial cell apoptosis, thus preventing endothelial dysfunction.

Furthermore, a limitation of the present study is the need for further *in vivo* pharmacodynamic studies and validation of other primary cellular mechanisms. It was recently proposed that increased mitochondrial permeability and disturbed mitochondrial dynamics lead to apoptosis and that mitochondria play important roles in regulating the Ca^2+^ concentration [63]. The relationship between mitochondrial dysfunction and endoplasmic reticulum stress is close, but the specific mechanisms involved in MGO‐induced apoptosis and the regulation of calcium ion expression need further investigation. Additionally, the levels of calcium channel‐associated proteins were measured via a qRT‐PCR assay, which was too homogeneous. Therefore, evaluation via protein level assays is warranted in our future studies. Although ACA is a promising candidate drug for the treatment of diabetic vascular disease, clinical pharmacological information on ACA is currently insufficient. However, ACA is undeniably a promising candidate for diabetic vascular disease. In summary, we found that ACA can effectively protect against MGO‐induced apoptosis in HUVECs. Specifically, ACA might inhibit calcium influx through MGO‐induced cells, upregulate phosphorylated eNOS (Ser1177) and NO, decrease the expression of ERS‐related proteins, and reduce endothelial cell apoptosis, thus preventing endothelial dysfunction (Fig. 7). These findings provide novel information on the benefits of ACA therapy and new insights into developing strategies to protect endothelial function in diabetic vascular disease.

## Conflicts of interest

The authors declare that they have no conflicts of interest.

## Author contributions

ZZ, DKY and GFM were responsible for study conception and design. ZZ was responsible for data collection. ZZ, KEH and JC were responsible for data analysis. ZZ was responsible for drafting the manuscript. ZZ, DKY and GFM were responsible for manuscript revision. CYZ, ZHF and SHW were responsible for assistance with data collection. All authors reviewed the results and approved the final version of the manuscript submitted for publication.

## Supporting information


**Fig. S2.** Affinity values for direct interactions between eNOS and acacetin.
**Fig. S1.** Characterization data of acacetin. (A) The chemical structure of acacetin. (B) The structure of acacetin was detected by NMR, which met the requirements of experiment. (C) The purity of acacetin was detected by HPLC with the method as follows: column: Ultimate XB‐C18 4.6*150 mm, 5 μm; column temperature: 35 °C; detection mode: UV332 nm; flow rate: 1.0 mL·min^−1^; sample dissolution: methanol + DMF; mobile phase: A‐acetonitrile, B‐0.1% phosphoric acid in water; gradient elution: A, 37%–47%, 15 min, 47%–90%, 3 min. (D) Caculating the parameters of each peak from (C). The area of peak of acacetin in red frame is 98.84% represents that the purity of acacetin is 98.84%.

## Data Availability

The data that support the findings of this study are available from the corresponding author upon reasonable request.
