# Supplementary material for: Acacetin reduces endoplasmic reticulum stress through the P‐eNOS/PERK signaling pathway to attenuate MGO‐induced vascular endothelial cell dysfunction
Source: FEBS Open Bio. 2025 Feb 10;15(5):793–809. doi: 10.1002/2211-5463.70004 (PMC12051029; doi:10.1002/2211-5463.70004)
Supplement: Supplementary file 1 — Fig. S2. Affinity values for direct interactions between eNOS and acacetin. Fig. S1. Characterization data of acacetin. (A) The chemical structure of acacetin. (B) The structure of acacetin was detected by NMR, which met the requirements of experiment. (C) The purity of acacetin was detected by HPLC with the method as follows: column: Ultimate XB‐C18 4.6*150 mm, 5 μm; column temperature: 35 °C; detection mode: UV332 nm; flow rate: 1.0 mL·min−1; sample dissolution: methanol + DMF; mobile phase: A‐acetonitrile, B‐0.1% phosphoric acid in water; gradient elution: A, 37%–47%, 15 min, 47%–90%, 3 min. (D) Caculating the parameters of each peak from (C). The area of peak of acacetin in red frame is 98.84% represents that the purity of acacetin is 98.84%. [file FEB4-15-793-s001.docx]

**Acacetin reduces endoplasmic reticulum stress through the P-eNOS/PERK signaling pathway to attenuate MGO-induced vascular endothelial cell dysfunction**

Zhen Zhang^1^, Kaien Hu^1^, Zhaohui Fang^3^, Sihai Wang^3^, Jie Chen^1^, Dengke Yin^1*^, Caiyun Zhang^1*^, Gefei Ma^1,2*^

1. School of Pharmacy, Anhui University of Chinese Medicine, Hefei, 230012 Anhui Province China
2. Anhui Qimen Institute of Snakebite, Huangshan, 245000, P. R. China
3. Department of Endocrine, the First Hospital Affiliated to Anhui University of Chinese Medicine, No. 117 Meishan Road, Hefei 230038, Anhui, China.

* Corresponding authors

Email address: [yindengke@ahtcm.edu.cn](mailto:yindengke@ahtcm.edu.cn) (Dengke Yin); cyzhang@ahtcm.edu.cn (Caiyun Zhang); magefei@ahtcm.edu.cn (Gefei Ma)

Supplementary **Fig. S1.** Affinity values for direct interactions between eNOS and acacetin

| Mode\| affinity\| dist from best mode\| (kcal/mol)\| rmsd l.b.\| rmsd u.b. | | | |
| --- | --- | --- | --- |
| 1 | -9.4 | 0.000 | 0.000 |
| 2 | -9.3 | 11.800 | 16.300 |
| 3 | -9.2 | 3.381 | 6.757 |
| 4 | -8.6 | 16.304 | 17.982 |
| 5 | -8.6 | 16.443 | 18.319 |
| 6 | -8.4 | 10.847 | 12.890 |
| 7 | -8.4 | 19.605 | 22.938 |
| 8 | -8.0 | 11.623 | 12.679 |
| 9 | -7.8 | 23.207 | 25.076 |
| 10 | -7.8 | 11.957 | 14.101 |
| 11 | -7.7 | 11.345 | 12.204 |
| 12 | -7.7 | 12.164 | 14.308 |
| 13 | -7.7 | 20.508 | 22.772 |
| 14 | -7.5 | 23 711 | 25.699 |
| 15 | -7.5 | 12.683 | 14.732 |
| 16 | -7.4 | 13.545 | 15.400 |
| 17 | -7.4 | 24.431 | 25.516 |
| 18 | -7.3 | 12.142 | 14.152 |
| 19 | -7.2 | 12.504 | 14.175 |
| 20 | -7.0 | 22.115 | 22.796 |


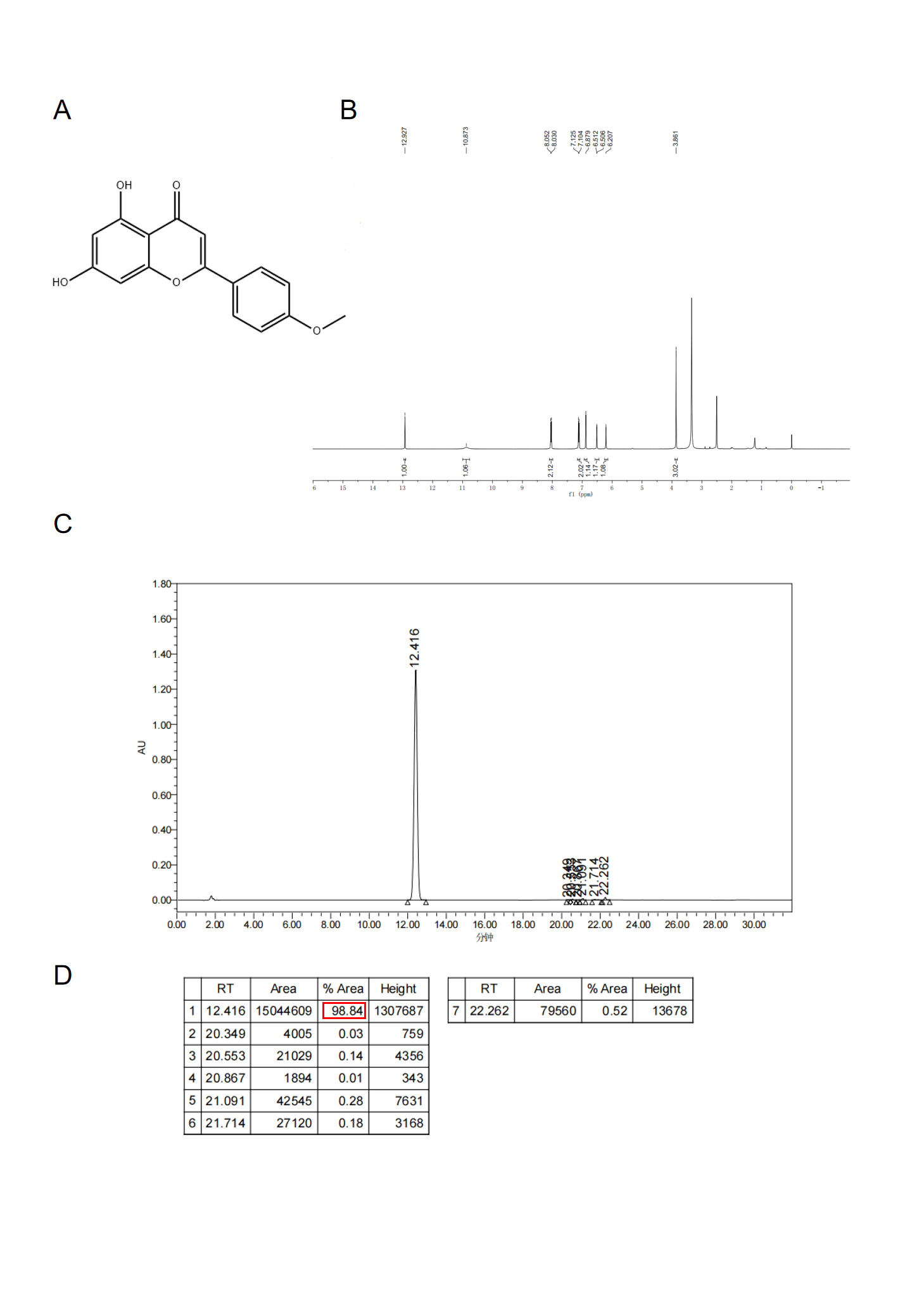


Supplementary **Fig. S2.** The characterization data of acacetin. (A) The chemical structure of acacetin. (B) The structure of acacetin was detected by Nuclear Magnetic Resonance, which met the requirements of experiment. (C) The purity of acacetin was detected by High Performance Liquid Chromatography with the method as follows: Column:Ultimate XB-C18 4.6*150mm,5μm; Column temperature: 35℃; Detection Mode: UV332 nm; Flow Rate:1.0ml/min; Sample dissolution:Methanol+DMF;Mobile Phase:A-Acetonitrile ,B-0.1% Phosphoric acid in water; Gradient elution:A,37%-47%,15min,47%-90%,3min. (D) Caculating the parameters of each peak from (C). The area of peak of acacetin in red frame is 98.84% represents that the purity of acacetin is 98.84%.
